# Effects of Ultrasonic Power on the Structure and Rheological Properties of Skin Collagen from Albacore (*Thunnus alalunga*)

**DOI:** 10.3390/md22020084

**Published:** 2024-02-10

**Authors:** Hao Pan, Xuehua Zhang, Jianbo Ni, Qianqian Liang, Xin Jiang, Zihui Zhou, Wenzheng Shi

**Affiliations:** 1 College of Food Sciences & Technology, Shanghai Ocean University, Shanghai 201306, China; panhaogongzuo@163.com (H.P.);; 2 Pingtairong Ocean Fisheries Co., Ltd., Zhoushan 316100, China; 3 National Research and Development Center for Processing Technology of Freshwater Aquatic Products (Shanghai), Shanghai 201306, China

**Keywords:** ultrasound-assisted extraction, collagen, extraction yield, structure, rheological properties, albacore skin

## Abstract

The effects of ultrasonic power (0, 150, 300, 450, and 600 W) on the extraction yield and the structure and rheological properties of pepsin-soluble collagen (PSC) from albacore skin were investigated. Compared with the conventional pepsin extraction method, ultrasonic treatment (UPSC) significantly increased the extraction yield of collagen from albacore skin, with a maximum increase of 8.56%. The sodium dodecyl sulfate-polyacrylamide gel electrophoresis analysis revealed that peptides of low molecular weight were produced when the ultrasonic power exceeded 300 W. Meanwhile, secondary structure, tertiary structure, and X-ray diffraction analyses showed that the original triple helix structure of collagen was intact after the ultrasonic treatment. The collagen solutions extracted under different ultrasonic powers had significant effects on the dynamic frequency sweep, but a steady shear test suggested that the collagen extracted at 150 W had the best viscosity. These results indicate that an ultrasonic power between 150 and 300 W can improve not only the extraction yield of natural collagen, but also the rheological properties of the collagen solution without compromising the triple helix structure.

## 1. Introduction

As the most abundant structural protein in the extracellular matrix of animal connective tissues (e.g., skin, bone, ligament, tendon, and cartilage), collagen plays a supporting and protective role in tissues. Of the 28 different types of collagen that have been identified and described so far, type I collagen is the dominant type in the skin [1]. Currently, collagen is still extracted from pigs and cows for commercial production, but its application is greatly limited due to the risk of disease transmission and religious beliefs [2,3]. Nevertheless, because of its useful function, collagen is needed in various fields such as food, medicine, biopharmaceuticals, cosmetics, etc., and the demand for collagen has been increasing [4,5,6]. Therefore, fish-derived collagen with low immunogenicity, non-toxicity, and fewer religious restrictions has attracted wide attention [7].

The traditional extraction methods for collagen include salt, acid, and pepsin extraction [8]. Currently, the pepsin extraction method is commonly used, where the Schiff base in the raw material is disrupted in an acidic environment, causing the collagen fibers to dissolve, and then proteinase can cut the terminal peptide region of the collagen triple helix, promoting the dissolution of collagen into the solution to improve the yield [9]. Ultrasonic is a physical green processing technology; the cavitation phenomenon leads to a large shear force in the medium, resulting in fragmentation, erosion, sonoporation, particle breakdown, and other effects, improving the extraction rate of various bioactive substances [10]. When ultrasound propagates in the liquid medium, the rapid generation and collapse of cavitation bubbles can enhance the penetration of the solvent into the solid matrix, thus achieving the effect of extraction [11]. Based on some recent research, the physicochemical, structural, and functional properties of golden carp [12], sea bass [13], sharpnose stingray [14], and soft-shelled turtle [15] were all significantly impacted by the application of ultrasonic technology in the collagen extraction process.

The albacore is one of seven highly profitable species of tuna, usually used as a raw material for processing, which generates a huge variety of by-products, with fish skin rich in collagen accounting for 10% of the whole fish. These by-products have generally been turned into fish meal by feed or pet food manufacturers, or even directly discarded [16]. Therefore, this experiment used albacore skin as its raw material to explore the impact of ultrasonic power on the yield, structure, and properties of collagen, providing a reference for the use of ultrasonic technology in the extraction of fish-derived collagen.

## 2. Results

### 2.1. The Effect of the Ultrasonic Power on the Collagen Yield

**Figure 1:**
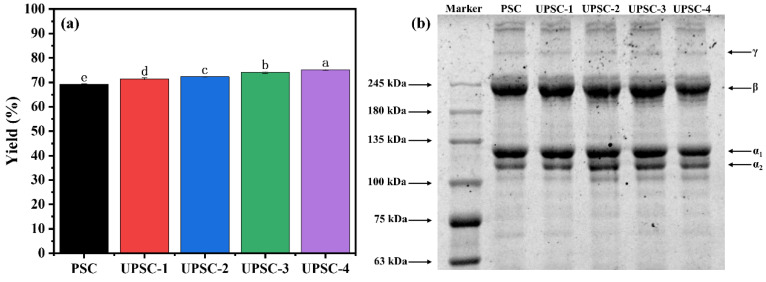
The yield (**a**) and SDS-PAGE profiles (**b**) of pepsin-soluble collagen-I from albacore skin extracted at different ultrasonic powers (non-ultrasonic treatment, PSC; 150 W, UPSC-1; 300 W, UPSC-2; 450 W, UPSC-3; 600 W, UPSC-4). Note: different letters in (**a**) indicate significant differences (*p* < 0.05).

The yield of the extraction of albacore skin collagen at different ultrasonic powers is summarized in Figure 1a. The ultrasound-assisted collagen extraction yield of UPSC of albacore skin was greater than the PSC yield (69.26%, dry weight) without ultrasonic treatment, and the collagen yield increased considerably with increasing ultrasonic power (*p* < 0.05). The maximum UPSC yield was achieved when the ultrasonic power was 600 W, reaching 75.18%. Similar research was executed by Ali et al. and Shaik et al., who found that ultrasound-assisted extraction has a positive impact on the yield of UPSC, possibly due to the cavitation effect that disrupts the cell wall, allowing pepsin to cleavage the telopeptide region of tropocollagen to a greater extent, and enhancing the solubilization of collagen in the skin matrix by acetic acid [12,14].

### 2.2. Amino Acid Composition Analysis

The amino acid content of albacore skin collagen under different ultrasonic power treatments is depicted in Table 1. Glycine (207–226/1000 residues) dominated the amino acids in the isolated collagen, which is crucial for the formation of triple helix structures, followed by glutamic (87–96/1000 residues), alanine (93–102/1000 residues), hydroxyproline (126–138/1000 residues), and proline (102–110/1000 residues) [17,18]. These results were comparable to the published amino acid content of collagen isolated from grass carp, yellowfin tuna, and Nile tilapia [7,19,20]. As the characteristic amino acids in collagen, the hydroxyproline (Hyp) and proline (Pro) content of UPSC increased first and then dropped with the increase in ultrasonic power, and was greater than that of PSC. The maximum imino acids (Hyp+ Pro) concentration of UPSC was 248 residues/1000 residues at an ultrasonic power of 300 W. Due to the high degree of damage to the skin matrix by high-power ultrasonic waves, other components can be extracted from the skin matrix, which causes a dilution effect and reduces the imino acid content of the excess collagen [21]. The imino acid content of the extracted collagen was similar to that of calf skin (216/1000 residues), pig skin (220/1000 residues), bigeye tuna (230–231/1000 residues), and squid skin (225/1000 residues) [22,23]. The stability of collagen is strongly tied to its imino acid content because its structure is maintained mostly by a pyridine ring, which regulates the conformation of the polypeptide chain, and partially by the creation of hydrogen bonds between hydroxyl groups on hydroxyproline [24]. Therefore, the UPSC-2 collagen extracted at an ultrasonic power of 300 W may have better thermal stability.

### 2.3. Sodium Dodecyl Sulfate-Polyacrylamide Gel Electrophoresis (SDS-PAGE) Analysis

Figure 1b shows the SDS-PAGE patterns of collagen isolated from albacore skin at various ultrasonic powers. All collagen samples contained α_1_ and α_2_, along with their dimer (β-chain) and a minor amount of trimer (γ-chain). According to the calculations of Image J (NIH, Bethesda, MD, USA), the strength proportion of α_1_ to α_2_ chains was roughly 2:1, which is in accordance with the type I collagen classification, and their molecular weights were 116 kDa and 125 kDa, respectively [8]. Albacore skin collagen’s SDS-PAGE patterns were identical to those of golden carp collagen, grass carp collagen, and Nile tilapia collagen [12,19,20]. Furthermore, as the ultrasonic power increased, a partial degradation of collagen occurred to some degree, with the development of peptides with molecular weights less than 110 kDa, notably at 300–600 W. This phenomenon was caused by the swelling of the albacore skin and the increased binding sites of pepsin at high ultrasonic powers. Petcharat et al. also observed a comparable occurrence; the α_1_ and α_2_ chains of collagen extracted from clown featherback skin degraded when the samples were treated with ultrasonic waves at 300 W or even higher powers [21].

### 2.4. Ultraviolet Spectrum (UV) Analysis

**Figure 2:**
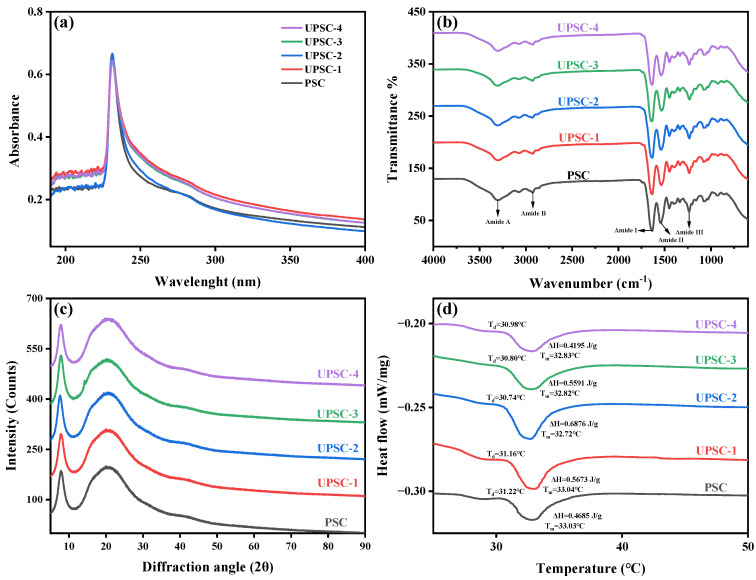
UV absorption spectra (**a**), FTIR spectra (**b**), XRD patterns (**c**), and DSC profiles (**d**) of pepsin-soluble collagen-I from albacore skin extracted using various ultrasonic powers.

The UV spectra changes in collagen isolated using various ultrasonic powers are shown in Figure 2a. The highest absorption peak was produced near 230 nm and was associated with carbonyl, carboxyl, and amide groups in the collagen polypeptide chains, which are characteristic absorption peaks related to the collagen’s triple helix structure [23]. All collagen samples exhibited an absorption peak at around 231.2 nm, which was consistent with the results observed in catla collagen, rohu collagen, lumpfish collagen, and starfish collagen [25,26]. Furthermore, a weak absorption peak was observed at 280 nm, revealing that the contents of tryptophan and tyrosine in the collagen were relatively low [27], which was compatible with the results of the above amino acid composition analysis.

### 2.5. Fourier Transform Infrared Spectroscopy (FTIR) Analysis

The FTIR of the collagens is depicted in Figure 2b. All the collagens’ amide A band (3300 cm^−1^) is associated with a N-H stretching vibration that normally arises within the range of 3440–3400 cm^−1^, and it was found that the band position commonly shifts to a lower frequency of about 3300 cm^−1^ when the N-H group of the peptide engages in hydrogen bonding. [28,29]. The amide B band of all collagen was located around 2928 cm^−1^, which is associated with the asymmetric stretching of CH_2_ and typically appears around 3080 cm^−1^ [30]. The amide I and amide II peaks of the infrared spectrum were related to the conformation of collagen and involved in the development of the collagen triple helix as a result of C=O stretching, N–H bending, and C–H stretching [31], which were not affected by ultrasonic power. The amide III band was discovered between 1238 and 1233 cm^−1^, which was the fingerprint spectrum of collagen, associated with a N-H deformation and C-N stretching vibration, which generally occurred in the region of 1350–1200 cm^−1^ and was responsible for the development of the collagen triple helix structure [32]. The triple helix conformation of collagen was usually maintained by restricting the change of the secondary conformation of the peptide chain in the pyrrolidone ring [7]. Furthermore, the absorption ratio between the amide III band and the CH_2_ bending was about 1.0, which proves that the triple helix conformation of the collagen samples from the albacore skin was still stable under different ultrasonic powers [33].

### 2.6. X-ray Diffraction (XRD) Analysis

As shown Figure 2c, the XRD patterns of the collagen exhibited two characteristic collagen peaks at diffraction angles (2θ) of 7.84° and 20.44°, respectively [19]. The triple helix structure of collagen was associated with a d value of 11.26 Å for the first peak, representing the distance between the molecular chains within the triple helix. The distance between the skeletons was reflected by the d value of 4.34 Å for the second peak, comparable to that of grass carp, Nile tilapia, and double-spotted pufferfish collagen [19,20,34]. These results were consistent with the diameter of the triple helix structure of the collagen molecule, indicating that the application of ultrasonic power had no effect on the natural triple helix conformation of collagen [20]. In summary, the extracted PSC and UPSC samples were confirmed to retain their original and intact triple helix structure through UV, FTIR, and XRD analyses.

### 2.7. Thermal Stability Analysis

Figure 2d shows the differential scanning calorimetry (DSC) curves of collagen under different ultrasonic powers. The ΔH of PSC, UPSC-1, UPSC-2, UPSC-3, and UPSC-4 was 0.4662, 0.5673, 0.6876, 0.5591, and 0.4195 J/g, respectively. The ΔH of UPSC was greater than that of PSC (*p* < 0.05), mainly due to its larger quantity of imino acids, especially hydroxyproline, which can provide hydroxyl groups during the formation of hydrogen bonds, showing a direct positive correlation with the thermal stability of proteins [35]. The pyrrolidone ring of proline and hydroxyproline are known to restrict the conformation of the peptide chain and contribute to the strengthening of the triple helix structure [36]. As the ultrasonic power increased, the ΔH of UPSC displayed a tendency to initially increase and then decrease, due to the high-intensity ultrasonic waves causing the fish skin to swell, increasing the binding sites of pepsin and resulting in different conformations of the peptide chains of the extracted collagen. Moreover, the T_max_ difference between UPSC and PSC was very small, which indicated that ultrasonic power itself did not alter the physical/chemical properties of collagen [12], comparable to the T_max_ of several tropical and subtropical species, including yellowfin tuna (33.7 °C), bigeye snapper (31.3 °C), and giant grouper (31.7 °C) [7,37,38]. On the other hand, the T_max_ of albacore skin collagen was significantly greater than that of temperate and cold-water species, including Spanish mackerel (15.12 °C), Japanese sea bass (26.5 °C), and deep-sea redfish (25 °C) [39,40,41]. However, the albacore skin collagen had a lower T_max_ than that of terrestrial species, such as calf skin (40.07 °C) [42].

### 2.8. Scanning Electron Microscopy (SEM) Analysis

**Figure 3:**
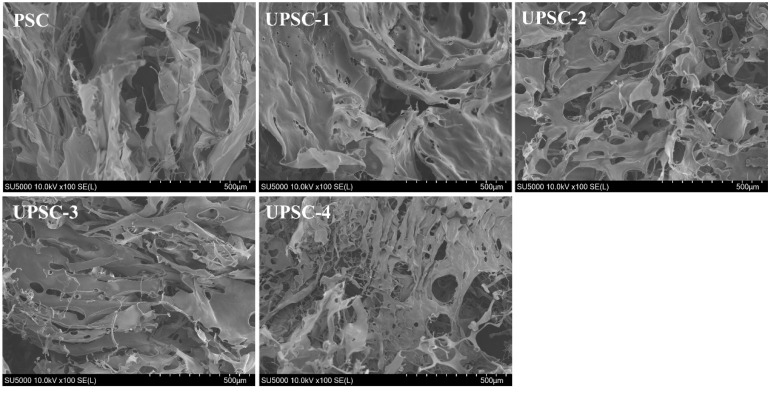
SEM images of pepsin-soluble collagen-I from albacore skin extracted at various ultrasonic powers, at 100× magnification.

The SEM images of the collagen extracted from albacore skin at different ultrasonic powers are shown in Figure 3. Scanning electron microscopy revealed that all collagens exhibited a similar surface morphology, with a loose fiber structure and irregularly twisted fiber ends connected by ripple sheets. Moreover, the PSC displayed a dense and multi-layered aggregation structure accompanied by surface wrinkles, potentially attributed to dehydration during the drying process [27]. However, the microstructure of the ultrasonically treated collagen showed a porous pattern, with a regular shape, similar to caves, which was induced by the cavitation and mechanical oscillation of the ultrasonic waves [15]. As the ultrasonic power increased, the microstructure of UPSC became looser, and its porous morphology became more obvious. These indicated that the microstructure of collagen extracted from albacore skin by ultrasound-assisted pepsin extraction had undergone obvious physical changes.

### 2.9. Rheological Properties

#### 2.9.1. Dynamic Frequency Sweep Tests

**Figure 4:**
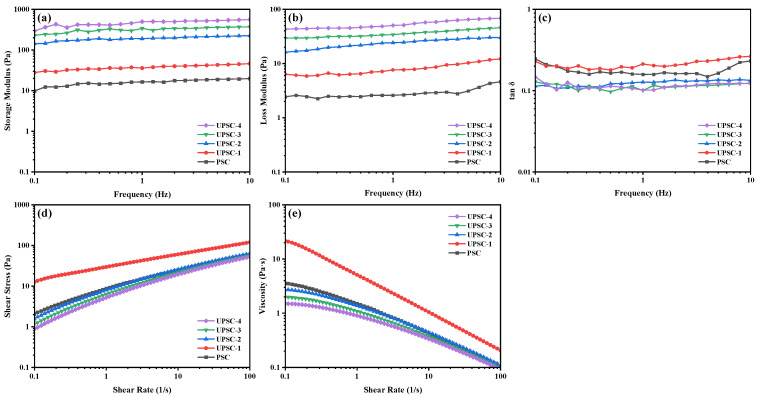
The storage modulus G′ (**a**), loss modulus G″ (**b**), and loss tangent tan δ (**c**) in dynamic frequency sweep test, and shear stress (**d**) and viscosity (**e**) in steady shear test of pepsin-soluble collagen-I from albacore skin extracted at different ultrasonic powers.

The frequency dependency of the storage modulus (G′), loss modulus (G″), and loss tangent (tan δ) was measured using the dynamic frequency sweep test. Figure 4a–c shows the dynamic frequency sweep curves of the collagen solutions extracted from albacore skin under different ultrasonic powers at 20 °C. It was discovered that there was a strong correlation between the storage modulus and the deformation of the polymer under external stress [43]. However, the G′ and G″ values of the collagen solutions which were extracted under different ultrasonic powers increased with the increasing frequency. When the ultrasonic power increased from 150 W to 600 W, the G′ and G″ values of the collagen solutions changed from 9.83 Pa and 2.43 Pa to 293.00 Pa and 43.10 Pa at 0.1 Hz, and from 19.90 Pa and 4.61 Pa to 559.00 Pa and 68.30 Pa at 10 Hz, respectively. It was noteworthy that the storage modulus of all collagen was higher than the loss modulus, demonstrating that the contribution of elasticity was greater than viscosity. The solution system mostly exhibits viscous behavior when tan δ > 1 (liquid-like behavior), and elastic behavior when tan δ < 1 (solid-like behavior) [44]. The smaller the tan δ value, the stronger the elasticity of the material [45]. Figure 4c shows the change of tan δ with frequency. It can be found that the tan δ values of all collagen solutions were less than 1, indicating that elasticity dominates the solution system, and the solution system shows solid-like behavior. These rheological characteristics resembled the collagen found in calf skin [44].

#### 2.9.2. Steady Shear Tests

Figure 4d,e show the curves of the shear stress and viscosity of albacore skin collagen solutions extracted under different ultrasonic powers at 20 °C as a function of the shear rate. The Ostwald-de Waele model can better describe the relationship between shear viscosity and shear rate, and the data fitted by the Ostwald de Waele model are shown in Table 2 (R^2^ > 0.97). The apparent viscosity significantly decreased as the shear rate increased, and the flow behavior index (*n* value) ranged from 0.3134 to 0.5819, indicating that all collagen samples exhibited shear-thinning pseudoplastic behavior [44,46]. The collagen solutions exhibited the typical shear thinning behavior of polymer solutions, with their viscosity decreasing with the increasing shear rate due to progressive chain orientation and untangling. As the ultrasonic power increased, the apparent viscosity of the collagen solutions increased from 3.58 Pa·s to 21.8 Pa·s and then decreased to 2.73 Pa·s. A possible explanation for this might be that the non-helical end peptide region at 150 W was removed by pepsin, resulting in a more ordered collagen structure, and an increase in the molecular interactions of the collagen, leading to increased chain entanglement and more pronounced non-Newtonian behavior. With the further increase in ultrasonic power, the structure of the collagen fibers was better opened, promoting the penetration and action of pepsin, leading to a partial degradation of collagen and thus a decrease in viscosity.

## 3. Materials and Methods

### 3.1. Materials

The albacore skin was provided by Zhejiang Pingtairong Ocean Fisheries Co., Ltd. (Zhoushan, China), with a moisture content of 60.03%, and stored at −20 °C. Other chemical reagents were all of analytical grade and were obtained from Shanghai Macklin Biochemical Co., Ltd. (Shanghai, China).

### 3.2. Extraction of Collagen

All operations were performed at 4 °C. The frozen albacore skin was immersed in 0.1 M NaOH solution (1:20, skin: NaOH solution, *w*/*v*) for 24 h; the NaOH solution was changed every 12 h. Thereafter, the skin was washed with pre-cooled distilled water until the wash water reached pH 7.0. Next, the skin was soaked in butyl alcohol solution (10%, *v*/*v*) at a ratio of 1:20 (*w*/*v*) for 24 h; the butyl alcohol solution was changed every 12 h. Then, the defatted sample was repeatedly washed with pre-cooled distilled water to remove excess butyl alcohol. The cleaned skin was cut manually into small pieces of 0.5 × 0.5 cm^2^ to facilitate the extraction process.

Conventional pepsin-soluble collagen was called non-ultrasonic-treated pepsin-soluble collagen (PSC, control). The PSC was determined from albacore skin by using an older method with some modifications [20]. The pre-treated skin (8 g) was immersed in 0.05 M acetic acid (1:40, skin: acetic acid, *w*/*v*) containing 0.3% (*w*/*v*) pepsin for 30 min to swell partially, and then stirred for 48 h. The supernatant was obtained by centrifugation of the extraction solution (10,000× *g*) for 30 min, and then salted out with 2 M NaCl overnight. Furthermore, the solution was centrifuged at 10,000× *g* for 30 min, and the resulting precipitate was redissolved in 0.5 M acetic acid solution and dialyzed with 0.02 M Na_2_HPO_4_ solution for 24 h, 0.1 M acetic acid solution for 24 h, and finally with pre-cooled distilled water for 48 h, with the dialysate changed every 8 h. The resulting dialysate was lyophilized and then stored at −20 °C for subsequent analysis.

The pre-treated skin was soaked in 0.05 M acetic acid (1:40, skin: acetic acid, *w*/*v*) containing 0.3% (*w*/*v*) pepsin and ultra-sonicated at powers of 150, 300, 450, and 600 W for 30 min (off time 2 s and on time 2 s) using an ultrasonic processor with a probe (CP 750, Cole-Parmer, Shanghai, China) at frequency of 20 kHz, and labelled UPSC-1, UPSC-2, UPSC-3, and UPSC-4, respectively. After ultrasonic treatment, the UPSC was extracted via stirring for 48 h. The subsequent steps were performed as described above. The collagen yield was calculated using Equation (1):

(1) $$\mathrm{Yield}\;(\%)=\frac{lyophilized\;collagen\;weight}{albacore\;skin\;weight,dry\;basis}\times 100$$

### 3.3. Amino Acid Composition

The 20 mg lyophilized collagen sample was dissolved in 15 mL of 6 M HCl, evacuated with nitrogen, sealed, and hydrolyzed at 110 °C for 24 h. The hydrolysate was vaporized and the residue was dissolved in 25 mL of citric acid buffer solution. Analysis was carried out using an automatic amino acid analyzer (L-8800, Hitachi, Tokyo, Japan).

### 3.4. SDS-PAGE

The collagen solution (1 mg/mL) was mixed with the sample loading buffer at a ratio of 1:1 (*v*/*v*), and then denatured at 95 °C for 10 min. Twenty microliters of the collagen samples were loaded onto a polyacrylamide gel consisting of 6% separating gel and 5% stacking gel. Electrophoresis was performed on a Bio-Rad Mini-PROTEAN II System Cell instrument (Bio-Rad Laboratories, Hercules, CA, USA) at a constant voltage of 120 V for 40 min. The gel was stained with Coomassie brilliant blue R-250 for 30 min and then destained until the background became transparent. Following destaining, the gel was scanned using a gel imager (Universal Hood II, Bio-Rad, Hercules, CA, USA).

### 3.5. UV

The lyophilized collagen (0.5 mg/mL) sample was dissolved in 0.5 M acetic acid. The absorbance of the UV spectrum at 190–400 nm was recorded at a scanning rate of 0.2 nm/s and a temperature of 25 °C using a UV–visible spectrophotometer (UV-2600i, Shimadzu, Tokyo, Japan).

### 3.6. FTIR

The FTIR of the lyophilized collagen samples was analyzed using a FTIR imaging system (Spotlight 400, PerkinElmer, Seer Green, UK). The collagen samples were scanned 32 times in the spectral range of 4000–600 cm^−1^ with a resolution of 4 cm^−1^ at 25 °C.

### 3.7. XRD

The XRD pattern was scanned based on the method described by Yang et al. (2022) [19]. X-ray diffraction was used to analyze the collagen protein sample, utilizing an X-ray diffractometer (Ultima IV, Rigaku, Tokyo, Japan). CuKa was used as the X-ray source, with a 2θ range of 5–90° and a scanning speed of 2°/min.

### 3.8. Thermal Stability

The thermal stability of the collagen samples was evaluated utilizing a differential scanning calorimeter (Q2000, TA Instruments, New Castle, DE, USA). A lyophilized collagen sample was redissolved in deionized water (1:40, collagen sample: deionized water, *w*/*v*) and allowed to stabilize at 4 °C for 24 h. The differential scanning calorimeter was used as required, and temperature and heat flow were calibrated with indium before the experiment. An aluminum pan containing 5–10 mg of the sample was sealed using a press. After placing the sample in the DSC device, the system was first cooled to 20 °C, equilibrated for 10 min, and then the curve was recorded by rising from 20 °C to 70 °C with a heating speed of 5 °C/min.

### 3.9. SEM

The lyophilized collagen sample was taped to the sample holder and sputter-coated with gold. The microstructures were observed at a magnification of 100× and a 10 kV accelerating voltage using a scanning electron microscope (SU5000, Hitachi, Tokyo, Japan).

### 3.10. Rheological Properties

Dynamic frequency scanning tests and steady shear tests were determined using the protocol outlined in Zhang et al. with some modifications [47]. The rheological properties of collagen were conducted using a rheometer (MCR 301, Anton Paar, Baden-Wuerttemberg, Germany) with a stainless steel plate of PP50 geometry and a gap of 0.1 mm. Before measurement, the sample was stabilized at 20 °C for 10 min. The UPSC sample solution (10 mg/mL) was subjected to dynamic frequency scanning at 20 °C, with a 0.01 to 10 Hz frequency range and 1% constant strain. The steady-state viscosity curve of the UPSC sample solution (10 mg/mL) was calculated at 20 °C and described by the Ostwald de Waele model (Equation (2)) [19].

*η* = *Kγ*^*n*−1^ (2)

where *η* is the shear viscosity, *K* is the consistency index, *γ* is the shear rate, and *n* is the flow behavior index.

### 3.11. Statistical Analysis

Every indicator was measured at least three times. The data were provided in the form of the mean  ±  standard deviation (SD). One-way ANOVA and Duncan’s multiple range tests were implemented to determine significance (*p* < 0.05) using SPSS 26.0 software (IBM, Armonk, New York, NY, USA). Origin2023b software (Origin Lab Corporation, Northampton, MA, USA) was used for the graphs.

## 4. Conclusions

This study found that increasing the ultrasonic power boosted the collagen extraction yield significantly, and that the isolated collagens were all type I collagens with good purity. However, when the ultrasonic power exceeded 300 W, low-molecular-weight peptides were produced, accompanied by a decrease in the imino acid content, leading to reduced thermal stability. This may be related to the excessive ultrasonic power accelerating the penetration of acetic acid and pepsin, leading to an increase in the binding sites of pepsin. Moreover, the original triple helix structure of the collagen was not affected by the ultrasonic power, as shown by FTIR and XRD analyses. Furthermore, dynamic frequency scanning and steady shear tests evaluated the changes in the rheological behavior of the collagen solutions extracted under various ultrasonic powers, and it was found that all collagen solutions exhibited shear-thinning pseudoplastic behavior. Increased ultrasonic power resulted in significant increases in G′ and G″, with the maximum viscosity of the collagen seen in that extracted at 150 W. In conclusion, an ultrasonic treatment is an effective method to assist in the extraction of collagen from fish skin. When the ultrasonic power is controlled between 150 and 300 W, it not only increases the extraction yield of natural collagen, but also does not destroy its triple helix structure, and has an advantageous impact on the rheological properties of the collagen solution.

## Figures and Tables

**Table 1:** Amino acid contents of pepsin-soluble collagen-I from albacore skin extracted at different ultrasonic powers (non-ultrasonic treatment, PSC; 150 W, UPSC-1; 300 W, UPSC-2; 450 W, UPSC-3; 600 W, UPSC-4) (residues/1000 amino acid residues).

Amino Acids	PSC	UPSC-1	UPSC-2	UPSC-3	UPSC-4
Asp	50.99 ± 2.58 ^a^	46.32 ± 1.86 ^b^	50.34 ± 0.53 ^ab^	47.29 ± 3.29 ^ab^	46.76 ± 1.78 ^ab^
Thr	31.11 ± 1.58 ^a^	28.56 ± 1.21 ^c^	30.71 ± 0.22 ^ab^	31.20 ± 1.07 ^a^	28.74 ± 1.05 ^bc^
Ser	34.95 ± 1.76 ^a^	32.39 ± 1.17 ^a^	34.49 ± 0.49 ^a^	30.71 ± 5.70 ^a^	32.35 ± 1.30 ^a^
Glu	96.34 ± 4.61 ^a^	87.09 ± 3.81 ^c^	94.50 ± 0.75 ^ab^	89.79 ± 4.57 ^abc^	88.91 ± 2.73 ^bc^
Gly	226.43 ± 9.47 ^a^	211.84 ± 2.84 ^bc^	220.78 ± 0.96 ^ab^	218.61 ± 4.27 ^ab^	207.61 ± 4.8 ^c^
Ala	102.69 ± 5.05 ^a^	93.64 ± 2.55 ^c^	100.25 ± 0.19 ^ab^	99.11 ± 2.53 ^ab^	96.35 ± 0.64 ^bc^
Val	23.09 ± 0.91 ^a^	21.44 ± 0.25 ^a^	22.88 ± 0.76 ^a^	23.66 ± 2.23 ^a^	27.22 ± 7.51 ^a^
Met	6.73 ± 1.35 ^b^	9.47 ± 2.39 ^ab^	12.99 ± 0.90 ^a^	10.17 ± 4.59 ^ab^	11.66 ± 4.31 ^ab^
Ile	11.06 ± 1.37 ^a^	13.96 ± 5.36 ^a^	11.41 ± 1.95 ^a^	13.38 ± 3.51 ^a^	14.92 ± 1.32 ^a^
Leu	26.16 ± 1.33 ^a^	33.01 ± 4.42 ^a^	26.62 ± 0.84 ^a^	35.9 ± 12.91 ^a^	38.51 ± 10.28 ^a^
Tyr	2.10 ± 0.15 ^b^	8.13 ± 1.44 ^a^	1.79 ± 0.23 ^b^	11.92 ± 6.52 ^a^	10.91 ± 2.22 ^a^
Phe	21.19 ± 0.63 ^b^	35.34 ± 2.42 ^a^	21.47 ± 0.67 ^b^	18.86 ± 2.82 ^b^	19.91 ± 2.57 ^b^
Lys	35.25 ± 1.56 ^b^	47.82 ± 1.86 ^a^	35.60 ± 1.49 ^b^	32.88 ± 2.93 ^b^	46.35 ± 6.12 ^a^
His	7.54 ± 0.43 ^b^	16.75 ± 3.43 ^ab^	8.08 ± 1.04 ^b^	20.60 ± 12.64 ^a^	26.83 ± 5.32 ^a^
Arg	83.15 ± 3.49 ^a^	74.01 ± 3.39 ^b^	79.56 ± 1.41 ^ab^	74.79 ± 4.82 ^b^	74.00 ± 2.16 ^b^
Pro	105.84 ± 4.82 ^ab^	106.91 ± 1.17 ^ab^	109.98 ± 2.54 ^a^	110.54 ± 1.48 ^a^	102.84 ± 2.69 ^b^
Hyp	135.39 ± 10.43 ^ab^	133.3 ± 1.98 ^ab^	138.56 ± 3.77 ^a^	130.61 ± 5.68 ^ab^	126.12 ± 0.93 ^b^
Imino acids	241.23 ± 15.08 ^ab^	240.22 ± 2.58 ^ab^	248.54 ± 1.29 ^a^	241.15 ± 6.32 ^ab^	228.97 ± 2.08 ^b^

Note: different letters in the same row indicate significant differences (*p* < 0.05).

**Table 2:** Rheological parameters from the Ostwald de Waele model of collagen solutions.

Collagen	Ostwald de Waele Model		
	*n*	*K* (Pa·s^*n*^)	R^2^
**PSC**	0.4772	1.382929	0.9927
**UPSC-1**	0.3134	5.035006	0.9997
**UPSC-2**	0.5819	0.807421	0.9759
**UPSC-3**	0.5486	0.979941	0.9816
**UPSC-4**	0.5184	1.241938	0.9851

## Data Availability

The original data presented in the study are included in the article; further inquiries can be directed to the corresponding author.
